# Stepwise emergence of CO gas sensing response and selectivity on SnO_2_ using C supports and PtO_x_ decoration

**DOI:** 10.3389/fchem.2024.1469520

**Published:** 2024-10-03

**Authors:** Yong Hwan Kim, Seung Yong Lee, Yunseong Ji, Jeong Ho Lee, Dae Woo Kim, Byeongdeok Lee, Changhyun Jin, Kyu Hyoung Lee

**Affiliations:** ^1^ Department of Materials Science and Engineering, Yonsei University, Seoul, Republic of Korea; ^2^ Department of Chemical and Biomolecular Engineering, Yonsei University, Seoul, Republic of Korea; ^3^ Materials Science and Chemical Engineering Center, Institute for Advanced Engineering, Yongin-si, Republic of Korea; ^4^ Yonsei-KIST Convergence Research Institute, Seoul, Republic of Korea

**Keywords:** SnO_2_, gas sensor, CO gases, room temperature, nanocomposites

## Abstract

Room temperature gas sensing is crucial for practical devices used in indoor environments. Among various materials, metal oxides are commonly used for gas sensing, but their strong insulating properties limit their effectiveness at room temperature. To address this issue, many studies have explored diverse methods such as nanoparticle decoration or conductive support, etc. Here, we report the emergence of gas-sensing functionality at room temperature with improved CO gas selectivity on SnO_2_ nanoparticles through sequential steps by using amorphous carbon (a-C) support and PtO_x_ decoration. The SnO_2_ decorated on amorphous carbon shows enhanced gas adsorption compared to inactive gas sensing on SnO_2_ decorated carbon support. The higher V_o_ site of SnO_2_ on a-C induces gas adsorption sites, which are related to the higher sp^2^ bonding caused by the large density of C defects. The ambiguous gas selectivity of SnO_2_/a-C is tailored by PtO_x_ decoration, which exhibits six values of sensing responses (R_g_/R_a_ or R_a_/R_g_) under CO gas at room temperature with higher selectivity. Compared to PtO_x_/a-C, which shows no response, the enhanced CO gas sensing functionality is attributed to the CO adsorption site on PtO_x_-decorated SnO_2_ particles. This report not only demonstrates the applicability of CO gas sensing at room temperature but also suggests a strategy for using SnO_2_ and carbon compositions in gas sensing devices.

## 1 Introduction

The increasing presence of air pollution gases, originating from not only the exhaust systems of combustion engines in heavy industries and vehicles but also from emissions in public places and residential buildings, has raised public awareness of biological health risks (Wang et al., 2018; Hou et al., 2012; Utriainen et al., 2003; Jia et al., 2014; Devaraj et al., 2022; Yamazoe, 2005). In response to this phenomenon, many studies have successfully developed gas sensing devices capable of detecting gases at sensitivities under a few parts per million (ppm) using metal oxide (MO) semiconductors. Consequently, the research trend in advanced gas-sensing materials has focused on improving sensitivity and selectivity, as measured by significant electrical resistance changes before and after analyte gas exposure on the MO surface (Dey, 2018; Franco et al., 2022; Eranna et al., 2024; Simon et al., 2001; Ji et al., 2019; Zakrzewska, 2001; Singh et al., 2021; Chen et al., 2013; Choi et al., 2021; Kim M. Y. et al., 2023; Kim et al., 2023b). This is achieved through various methods such as doping, nano-structuring, and creating heterojunctions. While these developed materials exhibit higher gas sensing performance at high temperatures and require significant electric power, there is a growing demand for room-temperature operational chemiresistor gas sensors. These sensors can be applied to smart mobile devices or IoT applications that monitor toxic gases in indoor environments with low electric power and integrated systems. Despite the increasing market demand for gas sensors that operate at room temperature, metal oxides face challenges due to their large band gaps and the interference caused by the preferred adsorption of hydroxyl groups over target gases on the MO surface (Tang et al., 2022; Srinivasan et al., 2019).

Among the diverse metal oxide (MO) candidates, SnO_2_ is one of the most widely used materials for gas sensing applications. SnO_2_ is an n-type semiconductor with a wide band gap of more than 3.0 eV, commonly used in gas sensors and catalytic activities due to its unique physical and chemical properties (Sun et al., 2022; Chen and Lou, 2013; Li et al., 2020; Masuda, 2022). Therefore, SnO_2_ has been investigated in various forms, such as hydrothermal, sol-gel, electrospinning, and polyol techniques, all of which have demonstrated promising performance (Chen and Lou, 2013; Masuda, 2022; Liu et al., 2023; Tonezzer et al., 2019; Mei et al., 2014; Yin et al., 2019). However, high-temperature operational SnO_2_-based gas sensors face challenges due to the strong insulating properties resulting from the large band gap and the adsorption of -OH groups from unavoidable moisture in the air, which interferes with the adsorption of analyte gases on the SnO_2_ surface (Tang et al., 2022; Srinivasan et al., 2019; Shah et al., 2022; Duoc et al., 2021). Thus, developing room-temperature operational SnO_2_ gas sensors has become a significant goal. To overcome these limitations, many studies have attempted to mix SnO_2_ with higher conductivity elements or employ nano-structuring techniques. Nevertheless, the consistently low response at room temperature makes it difficult to determine gas selectivity for these modified materials, leading to a trial-and-error approach in the development of room-temperature operational gas sensing materials.

In this report, we sequentially modify SnO_2_ to function as a room-temperature gas-sensing material using amorphous carbon (a-C) and PtO_x_. We enhance gas adsorption on SnO_2_ by using a-C support with a higher concentration of carbon defects, which shows no response in SnO_2_/C samples. Subsequently, PtO_x_-decorated SnO_2_/a-C demonstrates a CO gas selectivity with six of response, which is higher than the response for other gases. This improvement is due to CO adsorption on PtO_x_-coated SnO_2_ nanoparticles and external charge carrier conduction from PtO_x_/SnO_2_ to a-C, as opposed to the lack of gas response from PtO_x_ nanoparticles on a-C. These results suggest a method for modifying MO particles for room-temperature gas sensing using a sequential strategy.

## 2 Materials and methods

### 2.1 Material synthesis

SnO_2_/a-C. The 34 mmol of D-glucose was dissolved in 170 mL of deionized water and stirred for 30 min. The mixed solution was placed in Teflon inner container and performed hydrothermal synthesis at 200°C for 12 h. After performing centrifuge from hydrothermally synthesized solution were dried in the vacuum oven overnight. The dried powder was conducted in a tube furnace filled with Ar atmosphere at 500°C to obtain the a-C. SnO_2_ decoration was again performed via hydrothermal synthesis with SnCl_4_⋅5H_2_O and a-C. The solution of SnCl_4_⋅5H_2_O and a-C in deionized water was stirred for 30 min and hydrothermal synthesis was performed at 200°C for 12 h and sequentially dried the centrifuged powder in the vacuum oven overnight. The obtained powder was calcined under an Ar atmosphere at 500°C for 1 h.

SnO_2_/C. SnO_2_ decoration on carbon was performed via hydrothermal synthesis using SnCl_4_⋅5H_2_O with nano-sized carbon (Sigma-Aldrich, nano powder carbon <100 nm) under 200°C for 12 h. The solution of SnCl_4_⋅5H_2_O and carbon nanoparticles in deionized water was stirred for 30 min, and hydrothermal synthesis was performed under 200°C for 12 h. The resulting powder was calcined under an Ar atmosphere at 500°C for 1 h.

PtO_x_/SnO_2_/a-C and PtO_x_/a-C. PtO_x_ decoration on the SnO_2_/a-C or a-C particles was carried out using microwave synthesis for 30 s with H_2_PtCl_6_∙H_2_O (Sigma-Aldrich, 99.9% trace metals basis) dissolved in deionized water. After drying the synthesized powder overnight in the oven, we obtained the PtO_x_/SnO_2_/a-C and PtO_x_/a-C powder.

### 2.2 Material characterization

Morphological measurements were conducted by scanning electron microscopy (SEM, JEOL-7800F, JEOL Ltd.) and transmission electron microscopy (TEM, JEM-F200, JEOL Ltd.). The crystal structure characterization was performed using X-ray diffraction (XRD, Smart Lab, Rigaku) with Cu Kα radiation. Chemical bonding states were analyzed via X-ray photoelectron spectroscopy (XPS, K-alpha, Thermo Fisher Scientific Co.) Raman spectroscopy (LabRam Aramis, Horiba Jovin Yvon) was utilized to confirm the carbon vibration mode. The water contact angles for a-C and carbon were measured by Attension Theta Lite (Biolin Scientific).

### 2.3 Evaluate the gas-sensing performance

A 2-probe electrode configuration was employed for the gas sensing analysis. SnO_2_/a-C, SnO_2_/a-C, PtO_x_/SnO_2_/a-C, and PtO_x_/a-C properties were uniformly dispersed in ethanol at identical concentrations and subsequently drop-cast onto gold electrodes positioned on an alumina substrate. The gas-sensing performance of the fabricated sensors was evaluated within a custom-built chamber equipped with mass flow controllers, maintaining a fixed flow rate of 500 standard cubic centimeters per minute using air as the carrier gas. The sensors were exposed to target gas concentrations ranging up to 20 ppm for 500 s, followed by a recovery period in air for 1,000 s at a controlled temperature of 30°C. The resistance values in air (R_a_) and upon exposure to the target gases (R_g_) were recorded, and the sensor response (R_g_/R_a_) or R_a_/R_g_) was determined by calculating the ratio of resistance values in ambient air to those under gas exposure conditions Gas sensing measurements were performed for various gases, including Com HCHO, acetone, p-Xylene, Benzene, NO_2_, and SO_2_.

## 3 Results

### 3.1 Emergence of room temperature gas sensing on SnO_2_ using amorphous carbon

**FIGURE 1:**
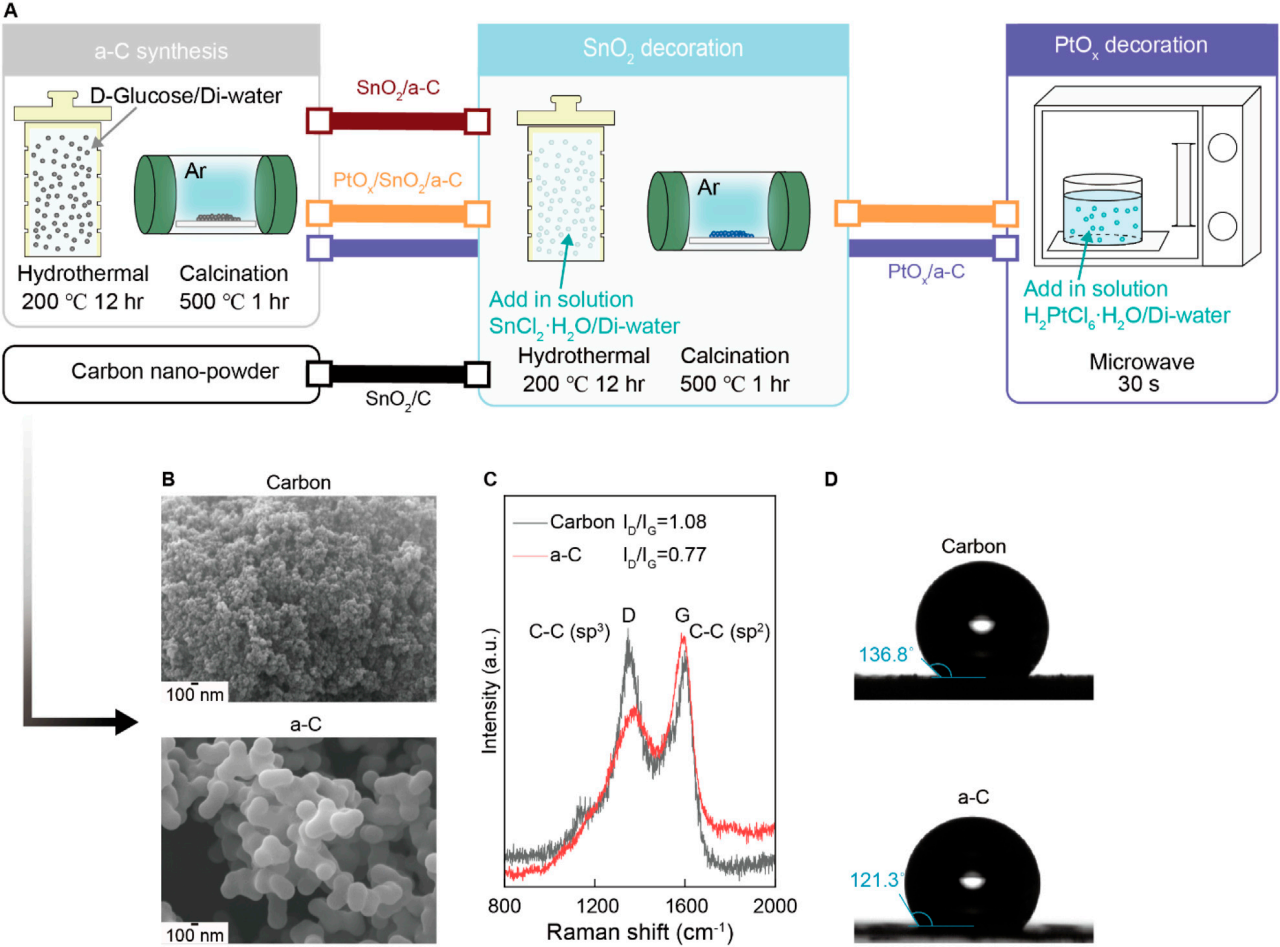
**(A)** Schematic illustration synthesis process of all samples. **(B)** SEM results of Carbon and a-C particles. **(C)** Raman results from Carbon and a-C particles. **(D)** Water contact angle difference of hydrophobic Carbon and a-C particles.

The emergence of gas adsorption sites on SnO_2_ nanoparticles depends on the bonding characteristics of the carbon supports. As shown in the SEM results in Figure 1A, we used various nano-sized carbon and a-C particles for support. The intensity ratio between sp^3^ carbon bonding at the D site and sp^2^ carbon bonding at the G site, noted as the I_D_/I_G_ ratio in the Raman spectra (Figure 1B), is 0.77 for a-C, indicating a higher G band (sp^2^ peak of carbon) bonding in a-C. This implies a higher C vacancy site than in the carbon particles (I_D_/I_G_ = 1.08) and suggests a higher -OH group concentration than in the carbon particles. Consequently, the favorable sp^2^ bonding character in a-C results in lower water contact angles compared to the carbon particle surface, as shown in Figure 1C.

**FIGURE 2:**
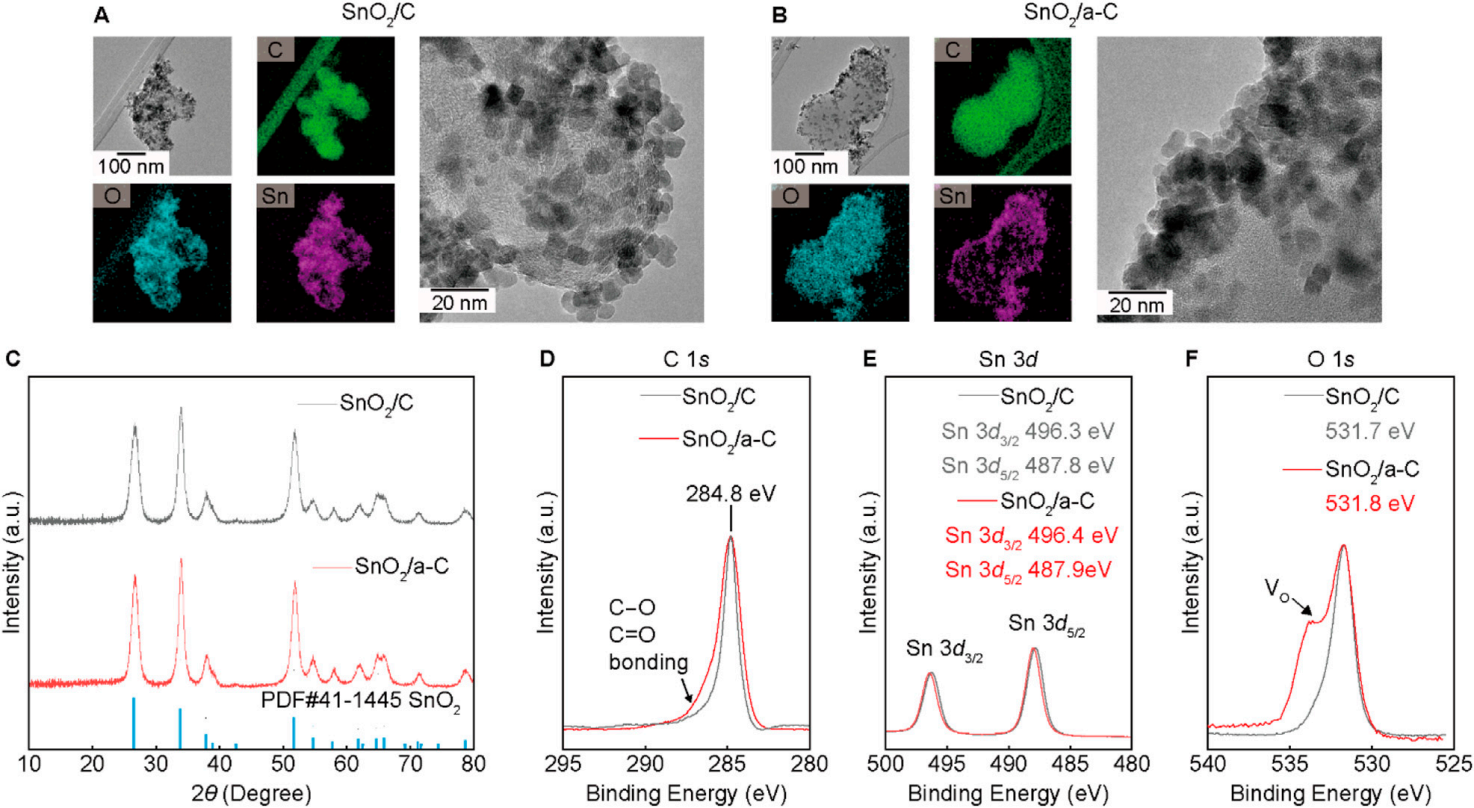
SnO_2_ on C or a-C particles. **(A, B)** TEM/EDS results of SnO_2_/C **(A)** and SnO_2_/a-C **(B)**. **(C)** XRD results of SnO_2_/C and SnO_2_/a-C. **(D–F)** XPS results with composed elements such as C 1*s* **(D)**, Sn 3*d* **(E)**, and O 1*s* **(F)**.

SnO_2_ nano-particles decorated on carbon supports (SnO_2_/C) and a-C (SnO_2_/a-C) are shown in the HR-TEM and EDS results (Figures 2A, B). Approximately 10 nm-sized SnO_2_ particles are evenly distributed on all Carbon supports. The X-ray diffraction (XRD) results in Figure 2C show the SnO_2_ single-phase peaks in all samples. However, the unobservable carbon peaks in the XRD results suggest lower crystallinity or an amorphous phase of the carbon supports. The XPS results for SnO_2_/C and SnO_2_/a-C (Figures 2D–F) demonstrate different chemical bonding characteristics. The carbon XPS results exhibit a larger asymmetric peak in SnO_2_/a-C binding energy. The remaining XPS intensity at higher binding energy than 284.8 eV indicates the existence of C-O single bonding or C=O double bonding, suggesting stronger bonding between a-C and Oxygen in SnO_2_ particles than in the Carbon supports (Kalish et al., 1999; Chokradjaroen et al., 2021). The Sn 3*d* and O 1*s* results display 496.4 eV for Sn 3*d*_3/2_, 487.9 eV for Sn 3*d*_5/2_, and 531.8 eV for O 1*s* in SnO_2_/a-C, whereas SnO_2_/C exhibits 496.3 eV for Sn 3*d*_3/2_, 487.8 eV for Sn 3*d*_5/2_, and 531.7 eV for O 1*s*. The higher binding energies of the peak positions indicate that the Sn on decorated SnO_2_ nanoparticles maintains the preferred +4 charge state on both the C and a-C supports, consistent with the single-phase SnO_2_ XRD pattern. Despite similar maximum peak positions for all samples, the higher Oxygen vacancy (V_O_) region compared to the symmetric shape of the O 1s peak is observed in the SnO_2_/C sample.

**FIGURE 3:**
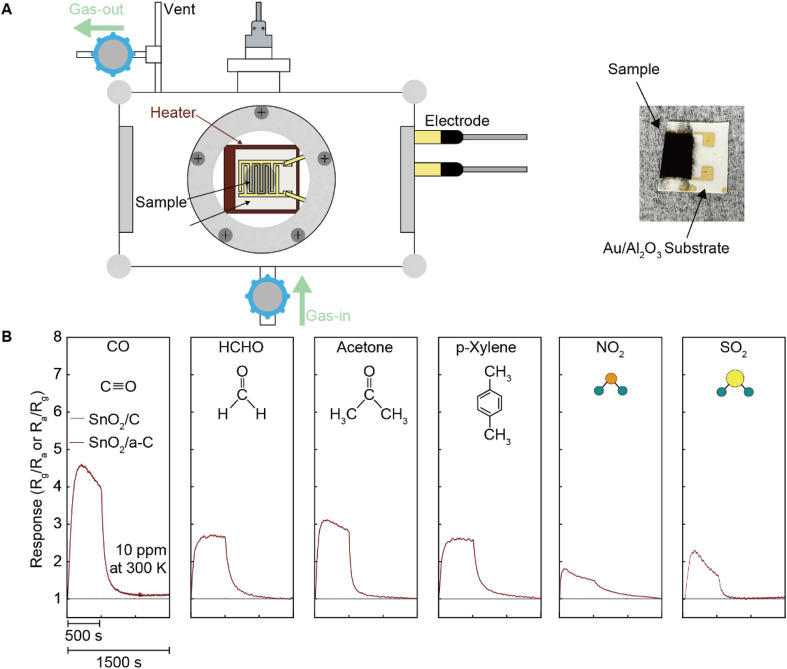
Emerged gas sensing functionality on SnO_2_/a-C than the SnO_2_/C at room temperature. **(A)** Schematically illustrated gas sensing measurement system and photo of SnO_2_/a-C coated Au/Al_2_O_3_ substrate. **(B)** Gas sensing is performed by resistance difference before/after diverse gases exposure such as CO, HCHO, acetone, p-Xylene, NO_2_, and SO.

The higher VO density in SnO_2_/a-C provides more activated gas adsorption sites compared to SnO_2_/C samples. The chemiresistive gas sensing functionality, shown in Figure 3, measured the electrical resistance differences before and after exposure to 10 ppm analyte gases (CO, HCHO, acetone, p-Xylene, NO_2_, and SO_2_) for SnO_2_/C and SnO_2_/a-C at room temperature. Unlike SnO_2_/C, which shows no significant electrical resistance difference under any of the analyte gases, SnO_2_/a-C exhibits a response unit (R_a_/R_g_, where R_a_ is the resistance in air and R_g_ is the resistance under analyte gas) of less than five for all gases. However, SnO_2_/a-C shows ambiguous gas selectivity, with a response of 4.5 for CO gas, which is higher compared to less than three for other gases.

### 3.2 Gas selectivity by PtO_x_ decoration on the SnO_2_/a-C

**FIGURE 4:**
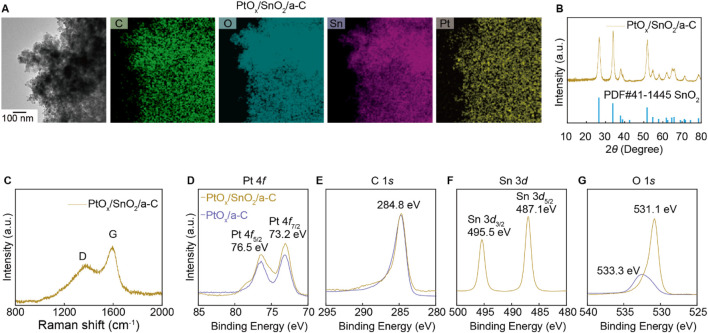
Structure analysis of PtO_x_ decorated on SnO_2_/a-C particles. **(A)** TEM/EDS mapping results. **(B)** XRD results. **(C)** Raman results. **(D–G)** XPS results of PtO_x_/SnO_2_/a-C and PtO_x_/a-C with composed elements such as Pt 4*f* **(D)**, C 1*s* **(E)**, Sn 3*d* **(F)**, and O 1*s* **(G)**.

The ambiguous gas selectivity of SnO_2_/a-C particles is modulated by decorating them with PtO_x_ nanoparticles using hydrothermal synthesis methods. The PtO_x_ nanoparticles are evenly distributed on the SnO_2_/a-C powder, as observed in the HR-TEM/EDS measurements in Figure 4A. The XRD results in Figure 4B show that the few wt% of PtO_x_ nanoparticles are decorated while maintaining the crystal structure of SnO_2_/a-C powder. The Raman spectra of PtO_x_/SnO_2_/a-C in Figure 4C show a higher sp^2^ vibration peak (G) compared to the sp^3^ vibration mode peak (D), indicating a higher carbon vacancy on the a-C support. The XPS results for PtO_x_/SnO_2_/a-C particles in Figures 4D–G show measurements for Pt 4*f*, C 1*s*, Sn 3*d*, and O 1*s*, respectively. Compared to SnO_2_/a-C, the PtO_x_/SnO_2_/a-C samples exhibit a higher binding energy region on a-C, indicating C-O and C=O bonding sites between SnO_2_ and a-C. The Sn and O measurements show peaks at 495.5 eV for Sn 3*d*_3/2_, 487.1 eV for Sn 3*d*_5/2_, and 531.1 eV for O 1*s*, respectively. The similar peak positions of the Sn 3*d* peaks indicate a consistent charge state of Sn in SnO_2_/a-C samples, remaining in the 4+ valence state. The lower V_O_ region in the O 1*s* XPS results after PtO_x_ decoration on SnO_2_/a-C suggests complex O bonding with Sn and Pt. The peaks at 73.2 eV for Pt 4*f*_7/2_ and 76.5 eV for Pt 4*f*_5/2_ indicate the fully ionized state of Pt cations as PtO_x_ on the SnO_2_ and a-C support. Furthermore, the oxidation state of Pt in PtO_x_/SnO_2_/a-C is similar to that in PtO_x_/a-C, independent of the matrix materials. However, the 533.3 eV O 1s peak, attributed to covalent bonding on the carbon tape, indicates a lower concentration of PtO_x_ nanoparticles, suggesting that they are physically decorated on the surface without strong chemical bonding with SnO_2_ or carbon. The gas selectivity is primarily generated by the PtO_x_ particles when they are decorated on SnO_2_ nanoparticles.

**FIGURE 5:**
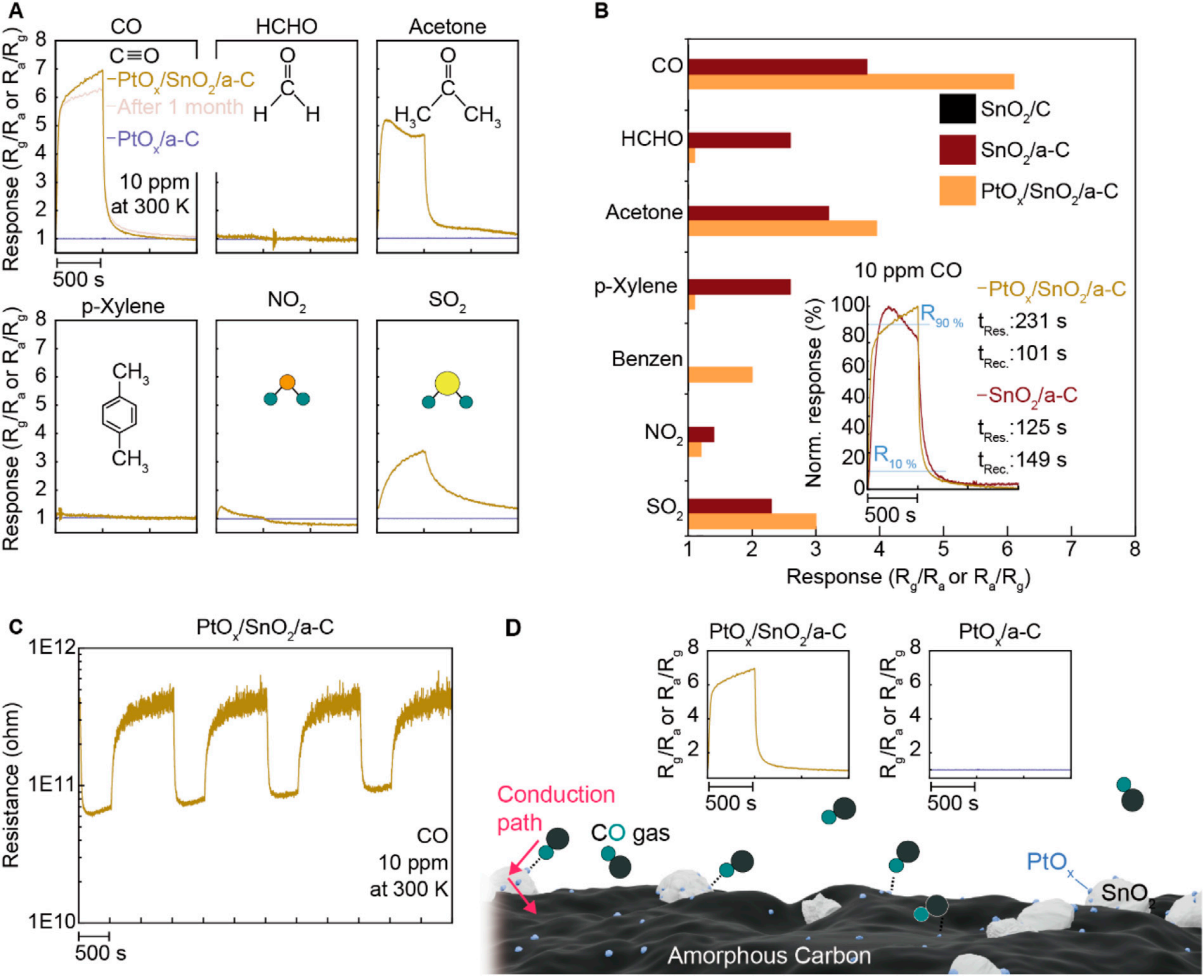
Specified gas selectivity on PtO_x_/SnO_2_/a-C. **(A)** Gas sensing response of PtO_x_/SnO_2_/a-C and PtO_x_/C under diverse gases such as CO, HCHO, acetone, p-Xylene, NO_2_, and SO_2_. **(B)** Comparison of PtO_x_/SnO_2_/a-C gas selectivity to SnO_2_/C and SnO_2_/a-C. The inset plot is the t_Res._ and t_Rec._ of PtO_x_/SnO_2_/a-C and SnO_2_/a-C under CO gas. **(C)** The resistance-time plot while operating the gas sensing test. **(D)** Schematic illustrated the emergence of higher CO analyte gas adsorption site on PtO_x_/SnO_2_/a-C by comparing with none of the gas sensing responses on PtO_x_/C.

To further investigate the role of PtO_x_, we conducted gas-sensing measurements under the same conditions (room temperature, 10 ppm concentration of CO, HCHO, acetone, p-Xylene, NO_2_, and SO_2_). We also synthesized PtO_x_/a-C particles and evaluated their gas-sensing performance. The results, shown in Figure 5A, reveal a gas-sensing response of nearly seven under CO gas. Additionally, the CO gas sensing response of PtO_x_/SnO_2_/a-C remained consistent 1 month after synthesis, demonstrating higher selectivity for CO compared to other gases (HCHO, acetone, p-Xylene, NO_2_, and SO_2_). In contrast, PtO_x_/a-C powder showed no gas-sensing functionality for all of the tested gases. Comparing the gas selectivity for SnO_2_/C, SnO_2_/a-C, and PtO_x_/SnO_2_/a-C, as plotted in Figure 5B, demonstrates that the decorated PtO_x_ particles impart CO gas selectivity to SnO_2_/a-C, which has an activated gas adsorption site. The response and recovery times (t_Res._ and t_Rec._) of SnO_2_/a-C and PtOx/SnO_2_/a-C under 10 ppm of CO gas are shown in the inset. The PtO_x_/SnO_2_/a-C sample displays a response time of 231 s and a recovery time of 101 s, while the SnO_2_/a-C sample exhibits a response time of 125 s and a recovery time of 149 s, respectively. A detailed analysis reveals that PtO_x_/SnO_2_/a-C demonstrates a faster t_Res._ than SnO_2_/a-C up to 70% of the maximum response, with the t_Res._ of PtO_x_/SnO_2_/a-C surpassing that of SnO_2_/a-C as it approaches the maximum gas response.

The reversible gas-sensing functionality of PtO_x_/SnO_2_/a-C during long-term gas-sensing measurements, shown in Figure 5C, demonstrates that PtO_x_/SnO_2_/a-C is an n-type gas-sensing device and exhibits a stable resistance range of 10^11^∼10^12^ Ω. The higher CO gas selectivity of PtO_x_/SnO_2_/a-C, compared to the lack of gas response on PtO_x_/a-C, demonstrates that PtO_x_ on SnO_2_ is the primary gas adsorption site, and PtO_x_-.SnO_2_-a-C provides a sequential external carrier conduction route, as described in Figure 5D.

## 4 Conclusion

Both a-C and PtO_x_ provide additional options for enabling gas sensing at room temperature on the SnO_2_ semiconductor. Specifically, a-C activates gas sensing functionality with higher gas adsorption at room temperature due to a higher concentration of carbon vacancies, while PtO_x_ on SnO_2_ induces higher CO gas selectivity. These results suggest that this strategy for developing gas sensing materials can be applied not only to SnO_2_ but also to other conventional metal oxides with large band gaps, enabling their operation at room temperature.

## Data Availability

The original contributions presented in the study are included in the article/Supplementary Material, further inquiries can be directed to the corresponding authors.
